# Conversion of specific lncRNAs to biomarkers in exhaled breath condensate samples of patients with advanced stage non-small-cell lung cancer

**DOI:** 10.3389/fgene.2023.1200262

**Published:** 2023-06-22

**Authors:** Aslı Tetik Vardarlı, Su Ozgur, Tuncay Goksel, Korcan Korba, Hardar Soydaner Karakus, Aycan Asık, Levent Pelit, Cumhur Gunduz

**Affiliations:** ^1^ Department of Medical Biology, Faculty of Medicine, Ege University, Izmir, Türkiye; ^2^ EgeSAM-Ege University Translational Pulmonary Research Center, Izmir, Türkiye; ^3^ Regional Hub for Cancer Registration in Northern Africa, Central and Western Asia, WHO/IARC-GICR, Izmir, Türkiye; ^4^ Department of Pulmonary Medicine, Faculty of Medicine, Ege University, Izmir, Türkiye; ^5^ Department of Chemical Engineering, Faculty of Engineering, Ege University, Izmir, Türkiye; ^6^ Department of Medical Biology, Faculty of Medicine, Mugla Sıtkı Kocman University, Mugla, Türkiye; ^7^ Department of Chemistry, Faculty of Science, Ege University, Izmir, Türkiye

**Keywords:** lung cancer, cfRNA, lncRNA, ebc, biomarker

## Abstract

**Objectives:** Lung cancer (LC) is one of the most prevalent cancers with the highest fatality rate worldwide. Long noncoding RNAs (lncRNAs) are being considered potential new molecular targets for early diagnosis, follow-up, and individual treatment decisions in LC. Therefore, this study evaluated whether lncRNA expression levels obtained from exhaled breath condensate (EBC) samples play a role in the occurrence of metastasis in the diagnosis and follow-up of patients with advanced lung adenocarcinoma (LA).

**Methods:** A total of 40 patients with advanced primary LA and 20 healthy controls participated in the study. EBC samples were collected from patients (during diagnosis and follow-up) and healthy individuals for molecular analysis. Liquid biopsy samples were also randomly obtained from 10 patients with LA and 10 healthy people. The expression of lncRNA genes, such as MALAT1, HOTAIR, PVT1, NEAT1, ANRIL, and SPRY4-IT1 was analyzed using cfRNA extracted from all clinical samples.

**Results:** In the diagnosis and follow-up of patients with LA, lncRNA HOTAIR (5-fold), PVT1 (7.9-fold), and NEAT1 (12.8-fold), PVT1 (6.8-fold), MALAT1 (8.4-fold) expression levels were significantly higher than those in healthy controls, respectively. Additionally, the distinct lncRNA expression profiles identified in EBC samples imply that decreased ANRIL–NEAT1 and increased ANRIL gene expression levels can be used as biomarkers to predict the development of bone and lung metastases, respectively.

**Conclusion:** EBC is an innovative, easily reproducible approach for predicting the development of metastases, molecular diagnosis, and follow-up of LC. EBC has shown potential in elucidating the molecular structure of LC, monitoring changes, and discovering novel biomarkers.

## Introduction

Lung cancer (LC) is among the most common causes of mortality worldwide, accounting for 18.0% of all cancer deaths. The latest global cancer data indicated that the cancer burden increased to 19.3 million new cases and 10.0 million cancer deaths in 2020 and accounting for 2.21 million new cases and 1.80 million cancer deaths (Ferlay et al., 2021). LC is divided into two main classes: small-cell lung cancer (SCLC) and non-small-cell lung cancer (NSCLC). SCLC is the fastest growing and metastasizing kind of LC, accounting for 10%–15% of all cases, and NSCLC represents for 85%–90%. Adenocarcinoma, squamous cell carcinoma, and large-cell carcinoma are the most prevalent NSCLC subtypes (Hirsch et al., 2017). The adenocarcinoma subtype constitutes 40% of this group. Two-thirds of patients with LC are detected at an advanced stage, limiting the surgical therapeutic options (Shah and Masters, 2020). Immunotherapy and targeted medicines have been developed based on histology and molecular analysis in recent years, which have shown clinical benefits in patients with NSCLC (Shroff et al., 2018). Despite the development of new treatments, 5-year survival rates ranged from 15% to 20%, depending on the cancer stages (Lung Cancer Survival Rates). Drug resistance, metastasis development, and recurrence are defined as major factors that prevent the patients’ survival. Therefore, the identification of molecular mechanisms of these factors has become the main goal nowadays (Shroff et al., 2018).

While cancer has traditionally been defined as a set of diseases caused by accumulating genetic mutations as major causes of neoplasia, this paradigm has now been expanded to include the disruption of epigenetic regulatory mechanisms commonly occurring in cancer (Gutschner and Diederichs, 2012). Similar to genetic changes, epigenetic events affect almost every stage of tumor development. Understanding the epigenetic changes associated with cancer onset, progression, and metastasis is critical to improving our ability to successfully diagnose, treat, and prevent.

Noncoding RNAs are involved in epigenetic regulation through transcriptional and posttranscriptional mechanisms (Ferreira and Esteller, 2018). Long noncoding RNAs (lncRNAs) play important cellular and physiological roles, such as chromatin dynamics, proliferation, differentiation, developmental regulation, and gene expression (Li et al., 2014). The specificity of lncRNA expression attests to its essential roles in regulating organismal function and repairing pathological processes (Smolarz et al., 2021). The lncRNA properties, such as tissue or cellular specificity and regulation of gene expression at transcriptional and posttranscriptional levels, suggest that lncRNAs may be important in the formation of malignant tumors. Additionally, some lncRNAs are regulated by oncogene products or cancer transformation suppressors, indicating that they indirectly fulfill tumorigenic functions (Huarte, 2015).

Current findings have implicated aberrantly expressed lncRNAs such as MALAT1, HOTAIR, PVT1, NEAT1, ANRIL, and SPRY4-IT1 in the development and progression of numerous malignancies, including LC. Additionally, these lncRNAs can be used as biomarkers for cancer diagnosis, prognosis, and follow-up (Gutschner and Diederichs, 2012; Hu et al., 2016; Sun et al., 2016; Wang et al., 2021; Zeng et al., 2021). Identification and characterization of lncRNAs have opened a new era in cancer management. lncRNAs can serve as cancer biomarkers for developing new diagnostic tools and the prediction of prognosis and as potential targets for new cancer treatment strategies. The expression of particular lncRNAs is a distinguishing feature of different tissues and even individual cells. Although only a few lncRNAs are typically found in the body, the majority of individuals have extremely high concentrations of them. The lncRNAs that are tissue- or cell-specific have drastically reduced levels of expression (Jiang et al., 2016).

Tumor tissue biopsies, which are considered the gold standard, are crucial for detecting molecular alterations that must be examined to make the correct diagnosis and treatment decisions throughout the disease progression. However, this type of biopsies has restrictions, as they are invasive and insufficient for revealing the real-time molecular structure and monitoring changes in disease. To repeat and monitor these molecular analyses, novel noninvasive analysis methods are required. In this context, liquid biopsy is the most promising technique. Liquid biopsy samples, the first noninvasive procedure, contain nucleic acids (cell-free DNA and RNA [cfDNA and cfRNA]) that are freely circulating outside the cell. Nowadays, cfDNA and cfRNA analyses are employed as a noninvasive tool for identifying multiple genetic alterations associated with cancer, which is, therefore, being recognized as an alternative material widely used for early cancer diagnosis and targeted treatment decisions (Sorber et al., 2017). Current advances in the analysis of lncRNA processes in liquid biopsies make these molecules important clinical targets for cancer treatment (Schmitt and Chang, 2016). The introduction of personalized treatment options and immunotherapy because of advancements in molecular oncology is the driving force behind efforts to improve molecular biomarker tests and liquid biopsy, specifically in detecting these biomarkers.

Exhaled breath condensate (EBC) samples are an additional noninvasive approach investigated in recent years to determine the real-time and repeatable molecular structure of LC. EBC is a sample matrix consisting of aerosolized droplets from the alveolar fluid that are further diluted in the distal and proximal respiratory tracts and collected by condensation during tidal breathing (Davis et al., 2012). Due to its compound composition, EBC can be used to diagnose LC. EBC samples can yield components, such as protein, DNA, RNA, and volatile organic chemicals. EBC biomarkers are found in the respiratory air because of direct breathing. They are also directly implicated in the metabolic activities of cancer cells and provide the opportunity for genomics, transcriptomics, epigenomics, proteomics, and metabolomics examinations (Campbell et al., 2008). With the advancements in technologies, genetic analysis studies can be conducted using EBC samples and liquid biopsies in LC (Tetik Vardarli et al., 2020). The concordance of DNA and RNA analysis results from the liquid biopsy and EBC samples demonstrates that EBC is a promising noninvasive approach for LC molecular diagnosis and follow-up. Therefore, this study determined whether the expression profiles of certain lncRNAs, which are candidates for new molecular targets in the diagnosis, follow-up, and individual treatment decisions of LC, could be detected in EBC samples. Moreover, the use of MALAT1, HOTAIR, PVT1, NEAT1, ANRIL, and SPRY4-IT1 expression levels detected in cfRNA from EBC samples was also investigated for the molecular diagnosis and follow-up of LC.

## Materials and methods

### Study population

This case-control study included 40 primary adenocarcinoma patients (stage IIIB or IV) with 1–3 synchronous metastases and without metastases, who were being followed up in the Chest Diseases Department of Ege University Medical School between 2021 and 2022. Adenocarcinoma was diagnosed in the Pathology Department of Ege University Medical School in Izmir, Türkiye. This study also included 20 age- and gender-matched healthy individuals. The control participants were recruited from healthy volunteers without a history of cancer. The difference between two independent means (two groups) approximation was used for sample size calculation. The effect size between the two groups (cancer patients and healthy individuals) was calculated to be 0.7146, and the *post hoc* power was achieved to be 0.82. Detailed demographic and clinical characteristics were recorded for patients and healthy controls. The Institutional Medical Investigation Ethics Committee accepted the study protocol (21-7T/27), and all participants provided written informed consent.

### Clinic sample collection

EBC samples (Visit 0) were taken from 40 patients diagnosed with advanced stage (III and IVB) lung adenocarcinoma (LA) and 20 healthy individuals in the control group. Furthermore, peripheral blood samples were obtained at Visit 0 from 10 randomly selected patients with LA and one of every two individuals in the healthy control group to demonstrate the correlation of lncRNA expression levels in cfRNA samples obtained from EBC with liquid biopsy samples. Furthermore, EBC (Visit 1) samples were also collected from 26 patients diagnosed with LA during the third month of the follow-up.

### EBC sample collection

EBC samples (2 mL) were collected from patients and placed in a DNA- and RNA-free 15-mL tube with a homemade glass EBC condenser, as described in our previous study (Tetik Vardarli et al., 2020).

### Liquid biopsy collection

Peripheral blood was taken from patients included in this study in tubes with 4 mL ethylenediaminetetraacetic acid. Blood samples were centrifuged at 2000 *× g* for 10 min at 4°C within 2 h, and plasma samples were separated and transferred to a new centrifuge tube.

### RT-PCR analysis

cfRNA was extracted from EBC and plasma specimens of patients and healthy individuals using Norgen Total RNA Purification Kit (Norgen Biotek Corp.) according to the manufacturer’s instructions. cfRNA concentrations were measured using a Nanodrop 1000 instrument (Thermo Scientific). Then, cfRNAs were reverse transcribed into cDNA using the Transcriptor First Strand cDNA Synthesis Kit (Roche Applied Science) according to the manufacturer’s protocols. The quantitative real‐time RT-PCR method was used to determine MALAT1, HOTAIR, PVT1, NEAT1, ANRIL, and SPRY4-IT1 lncRNA expression levels using RT^2^ SYBR Green qPCR Mastermix Kit (Roche) and gene-specific primers (Table 1) via LightCycler 480 Instrument II (Roche, Switzerland). GAPDH, a housekeeping gene, was used for normalization.

**TABLE 1:** lncRNA primer sequences.

**ANRIL**	F:5′-CCA TCA GAG GTA ACA GTA GAG AC-3′
	R:5′-GAG GCA GGA GAA TCG CTT G-3′
**MALAT1**	F:5′-CAG CAG TTC GTG GTG AAG ATA G-3′
	R:5′-GCC TCC TCC GTG TGG TTG-3′
**PVT1**	F:5′-ATA TGG ACT GTG ATG CGG AAG-3′
	R:5′-ATA ACC TGT GAT GAA CCA ATA AGC-3′
**NEAT1**	F:5′- TAC ACA GCG AGG CAC CAC-3′
	R: 5′-GTC AGC ACA GGA GCA GAG G-3′
**SPRY4-IT1**	F:5′-GAG GGG TTC TTA AAT AGG CAG C-3′
	R:5′-GAG GTT CTT TAA AAA CAG CCC A-3′
**HOTAIR**	F:5′- CTG ACT CGC CTG TGC TCT G-3′
	R:5′-CCG CCG TCT GTA ACT CTG G-3′

### Statistical analysis

In lncRNA expression analysis, cycle threshold (CT) values obtained from the LC480 device were uploaded to the web-based online GeneGlobe Data Analysis Center (https://geneglobe.qiagen.com/us/analyze) software, normalized by the GAPDH gene expression, applied log2 transformation, and paired comparison of the groups. It was carried out in the same software with the 2^−ΔΔCT^ method. After comparisons, those with significant lncRNA expression fold change ± two-fold and above and *p* < 0.05 with Student’s *t*-test were listed in the web software. Additionally, comparative heat maps of the groups and correlation between EBC and liquid biopsy were performed with linear regression analysis in GraphPad Prism v.9.2. The importance of differentiated lncRNA expression analyses during the diagnosis using EBC and in reflecting the clinical outcomes of patients with LC was evaluated. The concordance among EBCs was interpreted using clinical data.

## Results

### Epidemiological and clinical findings of patients

The study included 20 healthy participants (10 F and 10 M) who had never been diagnosed with cancer and 40 patients with LA (11 F and 29 M). The average ages of patients diagnosed with LA and healthy control groups were 61.98 ± 9.59 (range: 40–79) and 60.20 ± 6.85 (range: 42–72) years, respectively. The patient characteristics with clinical samples collected during the study are displayed in Table 2.

**TABLE 2:** Demographic and clinical characteristics of patients with lung adenocarcinoma and the control group.

		Lung adenocarcinoma *n* = 40 (%)	Healthy control *n* = 20 (%)
Gender	Female	11 (27.5)	10 (50.0)
	Male	29 (72.5)	10 (50.0)
Age (year)	Female	61.1 ± 07.3	57.8 ± 7.3
	Male	62.3 ± 10.4	62.6 ± 5.7
Diagnosis	Stage IIIB	18 (45.0)	
	Stage IV	22 (55.0)	
Treatment	CT	8 (20.0)	
	CT and IT	10 (25.0)	
	CT and RT	10 (25.0)	
	CT, RT and IT	7 (17.5)	
	IT	4 (10.0)	
	RT and IT	1 (2.5)	
No Metastasis		18 (45.0)	
Metastasis		22 (55.0)	
Oligometastases	1	10 (45.0)	
	2	7 (31.8)	
	3	5 (27.7)	
Location of Oligometastases	Lung	14 (54.6)	
	Bone	8 (31.2)	
	Brain	5 (19.5)	
	Liver	3 (11.7)	
	Surrenal	3 (11.7)	
	Plevra	2 (7.8)	
	Soft tissue	2 (7.8)	
	Kidney	1 (3.9)	
	MSL	1 (3.9)	
Treatment duration (weeks)	Female	11.59 ± 11.91	
	Male	15.46 ± 11.87	

CT, chemotherapy; IT, immunotherapy; RT, radiotherapy; MSL, mediastinal subcarinal lymphadenopathy.

cfRNAs were successfully extracted from plasma (for liquid biopsy) and EBC samples which were taken for molecular analysis from patients (Visits 0 and 1) and healthy individuals (Visit 0).

### lncRNA expression analyses

The qRT-PCR method was used to investigate the role of EBC materials in determining lncRNA expression levels in the diagnosis and follow-up of LA. MALAT1, HOTAIR, PVT1, NEAT1, ANRIL, and SPRY4-IT1 lncRNA gene expression levels were evaluated in cfRNA obtained from patients with LA and healthy individuals.

**FIGURE 1:**
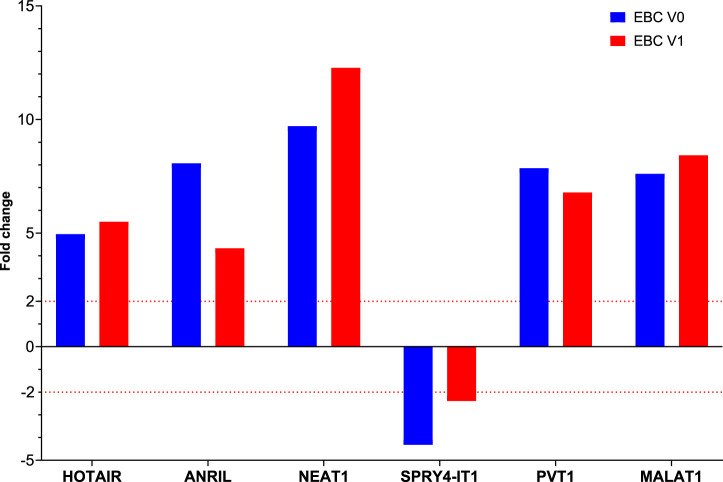
The fold changes in lncRNA expression levels were compared between EBC samples from patients at Visit 0 and Visit 1, and the control group.

Fold changes of specific lncRNAs associated with proliferation and metastasis in NSCLC were determined by comparing patients diagnosed with LA and the control group at Visits 0 and 1. These findings revealed the expression levels of some lncRNAs that function as oncogenes in the LC process, namely, HOTAIR, PVT1, NEAT1, and MALAT1, were significantly increased in EBC samples of the patient group. The lncRNAs showing the supreme increases at Visit-0 and Visit-1 in LA patients were identified as HOTAIR (5 fold, *p* = 0.004796), PVT1 (7.9 fold, *p* = 0.038149) and NEAT1 (12.8 fold, *p* = 0.009698), PVT1 (6.8 fold, *p* = 0.0493069), MALAT (8.4fold, *p* = 0.041893), respectively (Table 3; Figure 1).

**TABLE 3:** Fold changes of lncRNA expressions detected at Visits 0 and 1 compared with the control group in EBC samples.

	EBC visit 0		EBC visit 1	
lncRNA	Fold change	p	Fold change	p
HOTAIR	4.9588	0.004796	5.5008	0.085956
ANRIL	8.0700	0.101209	4.3349	0.306083
NEAT1	9.7075	0.154621	12.2778	0.009698
SPRY4-IT1	−4.3219	0.060601	−2.3959	0.397520
PVT1	7.8550	0.038149	6.7884	0.049306
MALAT1	7.6095	0.200276	8.4285	0.041893

**FIGURE 2:**
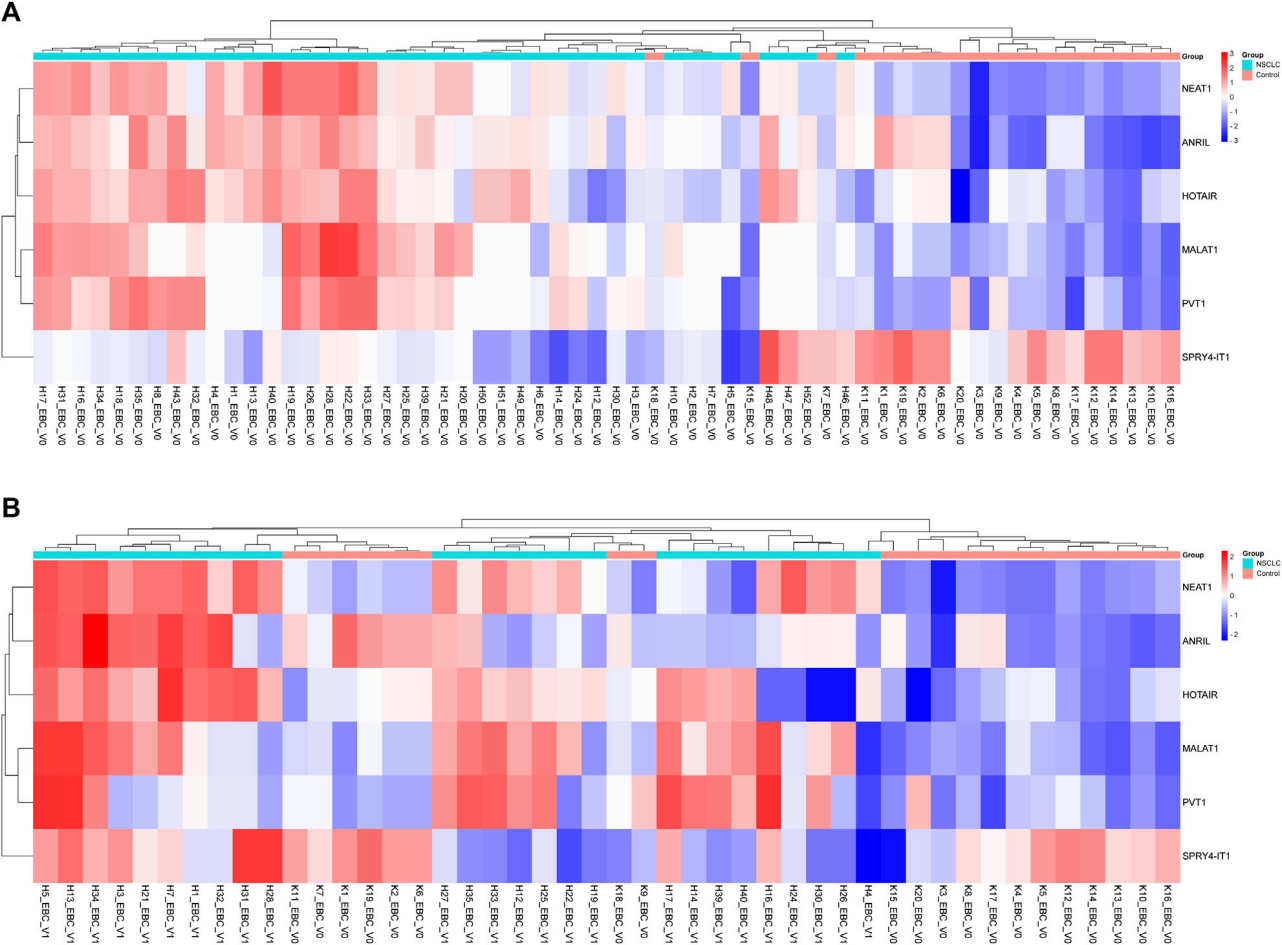
Displaying the expressions of lncRNAs in EBC samples of patients with NSCLC at Visits 0 and 1. **(A)** Illustration of a heatmap representing the 2-ΔΔCT changes in lncRNA expressions of Visit 0 EBC samples compared to the control group. **(B)** Displaying a heatmap representing the 2-ΔΔCT changes in lncRNA expressions of Visit 1 EBC samples compared to the control group.

To determine the roles of these lncRNAs in the follow-up of LA, specific lncRNA expression levels detected in EBC samples obtained from 26 patients diagnosed with LC at Visit 1 were compared with expression levels detected at Visit 0; our results reveal that NEAT1 expression level increased two-fold from Visit 0 to Visit 1 (*p* = 0.017159 [Table 4; Figure 2]).

**TABLE 4:** Fold changes of lncRNA expressions detected at Visit 1 compared with Visit 0 of EBC samples.

lncRNA	Fold change	p
HOTAIR	0.5460	**0.048808**
ANRIL	−3.6439	0.185442
NEAT1	2.5705	**0.017159**
SPRY4-IT1	1.9184	0.063224
PVT1	−1.0589	0.154379
MALAT1	0.8156	0.994734

The meaning of bold values emphasizes the *p*-values with significant.

**FIGURE 3:**
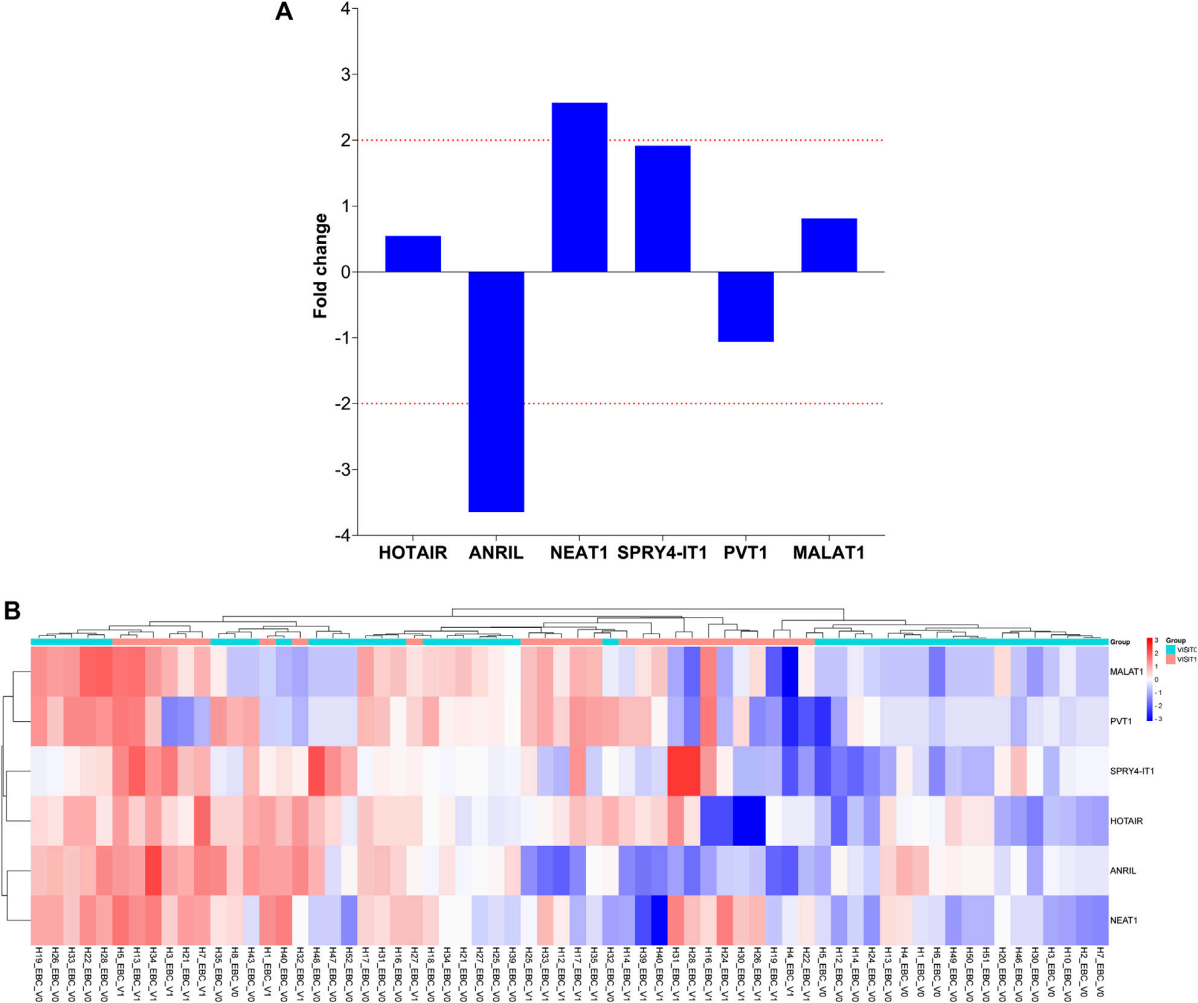
Illustration of the fold changes in lncRNA expression levels between Visit 1 and Visit 0 of EBC specimens. **(A)** Presenting a comparison of lncRNA expression levels at Visit 1 in EBC samples versus Visit 0. **(B)** Demonstration of a comparison of 2-ΔΔCT values for lncRNA expressions at Visit 1 in EBC samples compared to Visit 0.

To evaluate the significance of lncRNAs in LA development, the expression patterns of lncRNAs in plasma samples from 10 patients with LA and 10 healthy individuals with qRT-PCR were also investigated. When we examined the lncRNA expression levels at Visit 0, only the MALAT1 expression level obtained from the plasma samples was nine-fold higher in the patient group compared with the control group (*p* < 0.001 [Table 5; Figure 3]). To assess whether the lncRNA expression levels in EBC and plasma samples from patients with LC are consistent with one another, the expression levels found in EBC and plasma specimens were collected during Visit 0. However, no concordance was observed in the lncRNA expression levels between the two groups (*p* > 0.05).

**TABLE 5:** Fold changes of lncRNA expression levels at Visit 0 compared with the control group in plasma samples.

lncRNA	Fold change	p
HOTAIR	1.5311	0.262673
ANRIL	0.5558	0.274300
NEAT1	1.9855	0.337700
SPRY4-IT1	2.6781	0.730624
PVT1	8.4650	0.315780
MALAT1	8.9890	0.000001

Specific lncRNAs are highly dysregulated in various cancers and induce tumor progression and metastasis. The expression levels of specific lncRNAs in EBC samples taken from patients with LA with and without metastasis were compared at Visit 0 to evaluate the roles of MALAT1, HOTAIR, PVT1, NEAT1, ANRIL, and SPRY4-IT1 expression in the development of metastasis. Our findings showed that PVT1 expression levels decreased by two-fold in patients with organ metastasis.

**FIGURE 4:**
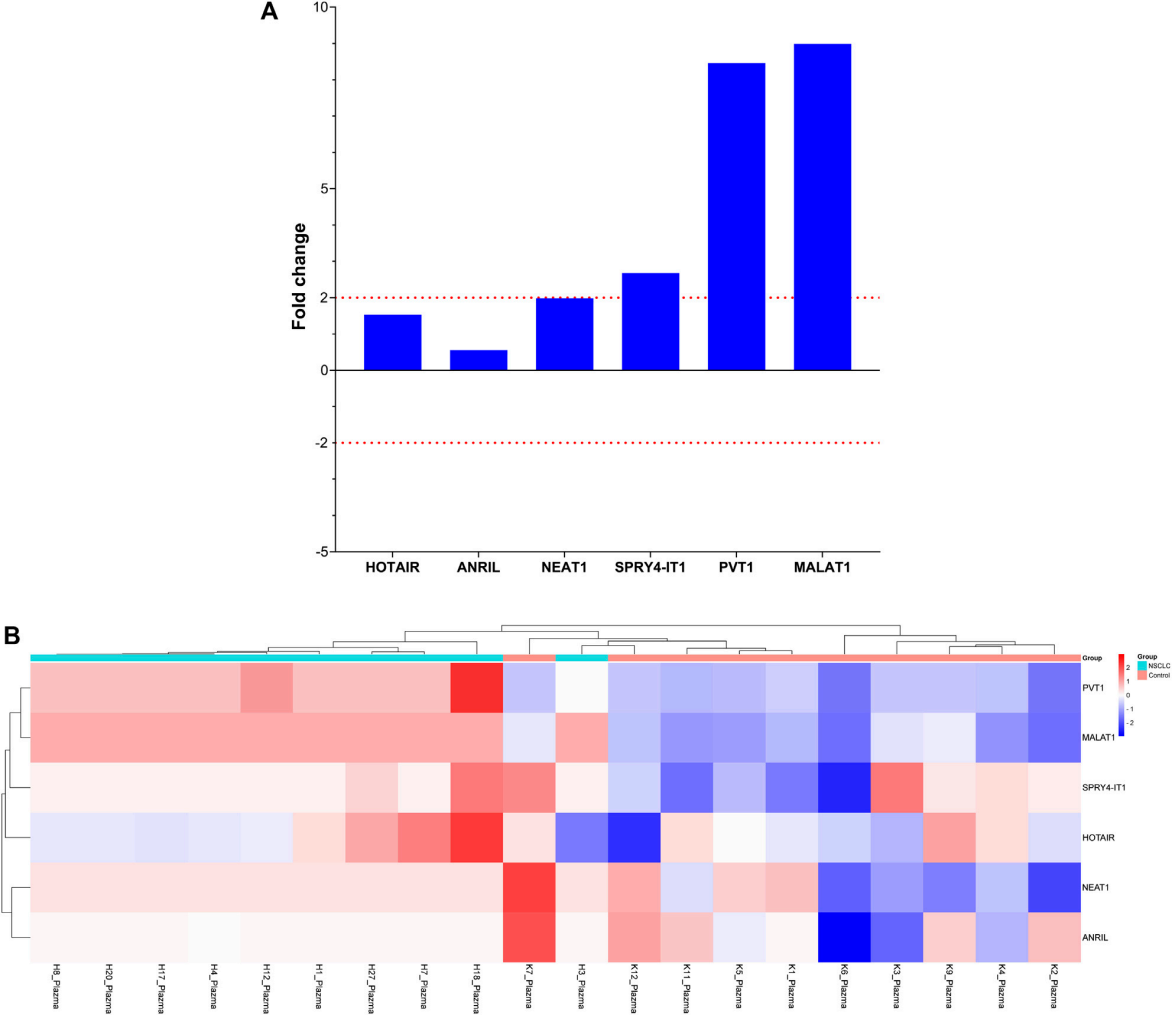
Depiction of the fold changes of specific lncRNAs in the plasma samples of patients with NSCLC at Visit 0. **(A)** Illustration of the fold changes in lncRNA expressions at Visit 0 of plasma cfRNA samples. **(B)** Displaying cases the 2-ΔΔCT changes in lncRNA expression of plasma cfRNA samples at Visit 0.

Comparison of lncRNA expression levels between patients with single or multiple organ metastases and those without metastasis revealed that HOTAIR expression level increased four-fold in patients with single organ metastasis and two-fold in those with multiple organ metastases, whereas PVT1 lncRNA expression levels decreased three-fold in those with multiple organ metastases. Changes in these lncRNA expression levels were found to be clinically significant in predicting the development of metastasis (Figure 4).

**FIGURE 5:**
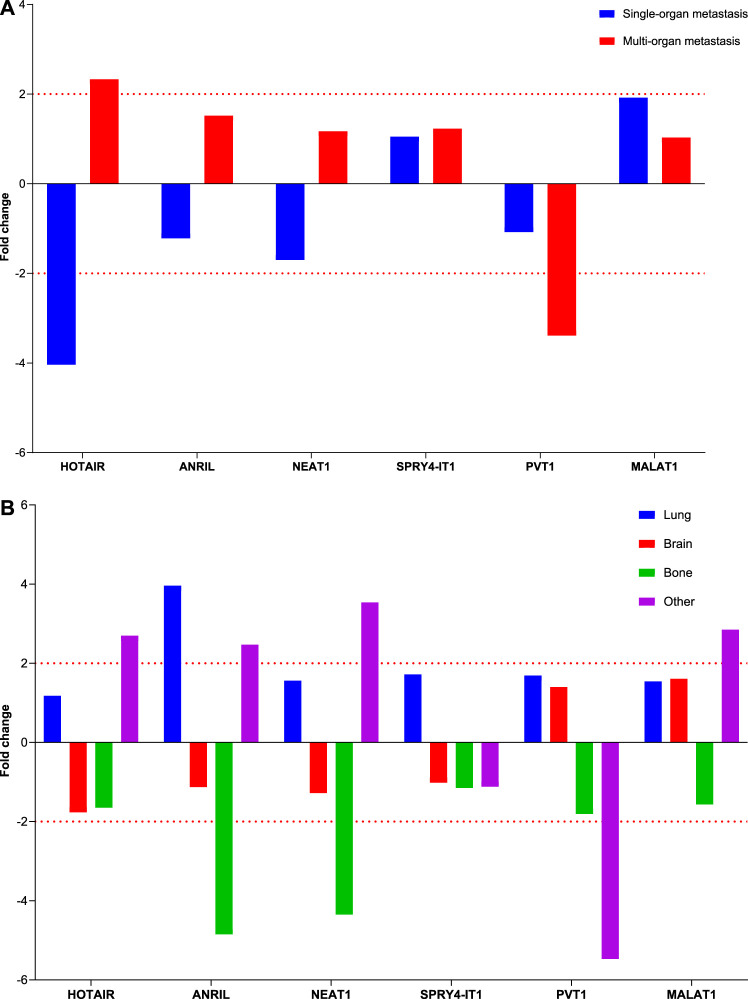
Illustration of the fold changes in lncRNA expressions in organ metastasis. **(A)** Depiction of the fold change of lncRNA expressions in patients with different organ metastases at Visit 0. **(B)** Displaying fold changes of lncRNA expression according to organ involvement.

Comparing the lncRNA expression levels at Visit 0 based on organ involvement in patients with various organ metastases to those without metastases revealed no variation in lncRNA expression levels among patients with brain metastasis. Interestingly, we found that ANRIL and NEAT1 gene expression levels were decreased four-fold in instances with bone involvement and elevated four-fold in those with lung involvement (Figure 5).

## Discussion

The study reveals that cfRNA extracted from EBC samples and liquid biopsy samples can be used to detect alterations in lncRNA gene expression levels for the molecular diagnosis and follow-up of LC. The development of new treatments that target molecular pathways implicated in carcinogenesis is encouraging; however, these treatments should effectively eliminate cancer cells without damaging healthy cells. To prevent difficulties associated with invasive procedures, early cancer diagnosis must depend on noninvasive, inexpensive, rapid, and reproducible techniques (You and Jones, 2012; Crowley et al., 2013; Sorber et al., 2017).

Several lncRNAs, such as MALAT1, HOTAIR, PVT1, NEAT1, ANRIL, and SPRY4-IT1, have recently been identified, particularly in cell lines, liquid biopsy, and tissue biopsy samples and have been associated with cancer pathogenesis. These findings suggest that lncRNAs provide new insights into the biology of this disease and could be used as a biomarker for early cancer detection. Consistent with other studies, we determined differentiated expression levels of lncRNAs in EBC samples from patients with LA. Additionally, HOTAIR and PVT1 (Visit 0) and NEAT1, PVT1, and MALAT1 (Visit 1) genes were overexpressed in EBC samples from patients with LA than those from healthy controls. The lncRNA gene expression profiles derived from the EBC sample can serve as both a diagnostic tool and a resource for patient follow-up. Increased HOTAIR gene expression levels have been identified as a diagnostic marker in patients with LA and lung squamous cell carcinoma in studies conducted with tumor tissue, plasma, and cell lines to determine the role of HOTAIR in NSCLC development (Wang et al., 2021; Yao et al., 2022). Yao et al. reported that HOTAIR expression is increased in LC tissues than in the control or surrounding normal tissues. Furthermore, they demonstrated an increased HOTAIR gene expression in both N and T stages. LncRNA HOTAIR has adequate diagnostic values for diagnosing LC (Yao et al., 2022). Similarly, HOTAIR gene expression was upregulated 5-fold and 5.5-fold in EBC samples from the patient group compared with the healthy control at Visits 0 and 1, respectively. HOTAIR seems to be an oncogene that plays a critical role in tumor progression. This study also shows that the HOTAIR gene expression profiles found in EBC samples are valuable for directing the molecular diagnosis of LC.

PVT1, an lncRNA, has been the focus of some preliminary studies in its potential function in NSCLC. PVT1 expression is higher in NSCLC tissue samples and cell lines, and PVT1 knockdown inhibits NSCLC cell proliferation, migration, and invasion (Wei et al., 2020; Wang et al., 2021). Additionally, Chen et al. revealed that A549 cells were made more sensitive to cisplatin after PVT1 was knocked down (Chen et al., 2019). Similarly, a higher expression pattern of the PVT1 gene in EBC samples of the patients was observed between both visits compared to healthy controls. We suggest that elevated PVT1 levels detected in EBC samples can be used as a diagnostic biomarker for NSCLC. These results lend support to the lncRNA analysis of EBC samples as a noninvasive method for detecting biomarkers in LA, as the lncRNA gene expression profile was shown to be similar to those of *in vivo* and *ex vivo* studies.

Several studies have profiled NEAT1 gene expression and its clinicopathological importance in NSCLC. Pan et al. reported that relative NEAT1 levels were considerably higher in NSCLC tissues than in nearby noncancerous lung tissues. Other studies also found a positive correlation between patient age and the presence of vascular invasion, lymph node metastases, and stage at diagnosis (Pan et al., 2015). Hu et al., (2016) reported that the levels of circulating NEAT1 were significantly higher in the plasma of patients with NSCLC. In a recent study that evaluated the effects of lncRNAs in plasma samples from 50 patients with NSCLC and 50 healthy individuals, researchers discovered 21 distinct lncRNAs and reported that SPRY4-IT1, ANRIL, and NEAT1 levels were significantly higher than those of other lncRNAs. It was emphasized that these three lncRNAs could serve as biomarkers for the early detection of NSCLC. Sun et al., (2016) also demonstrated that NEAT1 levels are elevated in patients with NSCLC, increasing cell proliferation and metastasis and inhibiting tumor cell death. Furthermore, they revealed that patients with elevated NEAT1 levels had lower overall survival than those with low NEAT1 gene expression, and that elevated NEAT1 levels accelerated NSCLC cell proliferation and metastasis *in vitro* and *in vivo*. Both the diagnosis and follow-up of patients with LA showed elevated NEAT1 gene expression levels, which is consistent with the findings of earlier studies. These data suggest that NEAT1 can be one of the recently found regulators of the NSCLC progression. The lncRNA MALAT1 is a type of lncRNA greatly overexpressed in many tumors, including NSCLC. However, the mechanism of MALAT1 in NSCLC remains unclear. Several studies have shown that MALAT1 is significantly upregulated in human NSCLC cell lines and patients with LC. The findings demonstrated that MALAT1 overexpression is closely associated with NSCLC malignancy, vascular invasion, pathological differentiation, and recurrence and can be used as a biomarker to diagnose malignant tumors (Weber et al., 2013; Rong et al., 2020). To assess MALAT1’s utility as a blood-based biomarker for NSCLC, Weber et al. compared the expression levels of MALAT1 in peripheral blood samples from patients with NSCLC and healthy individuals and discovered differences between the two groups (Weber et al., 2013). Rong et al., (2020) also suggested that MALAT1 levels in serum exosomes are higher in patients with NSCLC and that exosome-derived MALAT1 also reflect biological changes in NSCLC cells. Similarly, Zhang et al., (2017) reported that MALAT1 expression was upregulated in serum exosomes of patients with NSCLC and that exosomal MALAT1 level was positively correlated with tumor stage and lymph node metastasis. To demonstrate a correlation between variations in the lncRNA expression levels in EBC and the related plasma samples, lncRNA expression analysis was performed on liquid biopsy samples from both patients with LA and the control group. We found that the MALAT1 lncRNA expression was altered in both groups, although no concordance was noted in the expression levels. Liquid biopsy provides the opportunity to detect and monitor cancer in various body fluids by detecting free circulating tumor cells, circulating tumor DNA and RNA fragments, and exosomes (Sorber et al., 2017). It is advantageous in reducing the harm of biopsy by noninvasive sampling and is of great importance for the early detection of cancer; however, a study reported that the low lncRNA expression level in the blood may complicate sensitive analysis (Weber et al., 2013). We hypothesize that the late entry and reduced tumor cell expression in the blood circulation can cause difficulty in recognizing a significant correlation between the results. We estimate that the molecular profile obtained from EBC samples in patients diagnosed with NSCLC reflects the molecular profile of tissue specimens more accurately. These results suggest that MALAT1 gene expression levels in EBC samples, in addition to the tumor tissue, peripheral blood, and serum exosomes, can be used as a tumor biomarker for the diagnosis and follow-up of NSCLC.

Hu et al., (2016) reported that SPRY4-IT1 gene is overexpressed in the plasma of patients with NSCLC compared with healthy volunteers, and the amplification of SPRY4-IT1 in the plasma of patients with NSCLC is closely related to the tumor size. Additionally, SPRY4-IT1 can be used as a biomarker for the early diagnosis of NSCLC tumor cells with a poor prognosis because of epithelial–mesenchymal transition activation by regulating the E-cadherin and vimentin expressions. However, Sun et al. suggested that the SPRY4-IT1 expression is significantly downregulated in NSCLC tissues, and specifically, decreased SPRY4-IT1 expression may be an important independent predictive factor for patients with NSCLC. They also reported that the upregulation of SPRY4-IT1 expression significantly inhibited cell proliferation, migration, invasion, and apoptosis, whereas reduced expression of SPRY4-IT1 promoted cell migration and invasion. They revealed that increased SPRY4-IT1 expression levels significantly reduced the number of metastatic nodules in the lungs *in vivo* (Sun et al., 2016). Similar to this study, we also found that SPRY4-IT1 lncRNA expression levels decreased four-fold and three-fold in EBC samples obtained from patients during the diagnosis (Visit 0) and treatment (Visit 1) compared with healthy individuals, respectively. These data support the hypothesis that SPRY4-IT1 downregulation may serve as a possible diagnostic biomarker for NSCLC and may play various functions not only in the diagnostic but also in the follow-up process.

To determine the clinical significance and potential role of ANRIL, Nie et al., (2015) investigated the expression levels of ANRIL in tumor and healthy tissues of 96 patients with NSCLC. They showed that ANRIL expression was increased in tumor tissues and the expression level was significantly correlated with TNM stages and tumor size. Moreover, using loss-of-function analysis in NSCLC cells, studies demonstrated that suppressed ANRIL expression can inhibit cell proliferation and induce cell apoptosis both *in vitro* and *in vivo*. These findings revealed that the ANRIL expression is associated with survival in patients with NSCLC and is critical to NSCLC development. This study shows novel perspectives on the function of lncRNA-directed carcinogenesis. A study performed with LC and cervical cancer cells reported that silencing ANRIL stops the cell cycle at the G1/G0 checkpoint and leads the cell to apoptosis. Our data show that in EBC samples collected from patients during Visit 0 and 1, ANRIL gene expression levels were eight- and four-fold higher, respectively, than that in the control group. We also determined that ANRIL expression levels in the patient group decreased by three-fold during treatment. These findings revealed that ANRIL may be a significant biomarker of adenocarcinoma progression. Low ANRIL expression levels may be associated with a favorable prognosis, given that the survival of our patients persisted throughout the study duration and the identification of decreasing ANRIL gene expression levels during the follow-up period.

Current studies indicated that lncRNAs are closely related to metastasis cancer progression and formation of various cancer types. However, the molecular basis for this observation in NSCLC advancement has not yet been thoroughly described. Zeng et al., (2021) used primary tumor and adjacent tissues from 30 patients who had not received any local or systemic therapy preoperatively and LC cell lines to investigate how PVT1 regulates tumorigenesis and NSCLC development. They found that PVT1 is upregulated in NSCLC tissues and lung cell lines and promotes NSCLC cell proliferation, migration, invasion, and metastasis. Weber et al., (2013) demonstrated that MALAT1 knockdown in NSCLC confirmed its antiproliferation, antimetastasis, and proapoptosis activities in the loss-of-function analysis in NSCLC tumor tissue and cell line models, in addition to its tumor-promoting effects in the xenograft mouse model. Our results indicate that PVT1 expression levels were two-fold lower at Visit 0 in patients with LA with diverse organ metastases. Moreover, HOTAIR gene expression is downregulated four-fold in single organ involvement and upregulated two-fold in multiorgan involvement, whereas PVT1 gene expression is downregulated three-fold. Moreover, ANRIL gene expression increased four-fold in patients with lung involvement, whereas ANRIL and NEAT1 gene expressions decreased four-fold in patients with bone metastases. Based on these results, we hypothesize that PVT1, HOTAIR, ANRIL, and NEAT1 are useful biomarkers for monitoring the development of metastasis in patients with LA. LncRNAs, which are candidates for new molecular targets in determining the early diagnosis, follow-up, and individual treatment decisions of patients with NSCLC, can also be detected by EBC within the scope of this study. In lncRNA expression analysis conducted on EBC samples derived from patients with NSCLC and healthy controls, the expression profiles of MALAT1, HOTAIR, PVT1, NEAT1, ANRIL, and SPRY4-IT1 have been found, and an association between these expression profiles and molecular pathogenesis of LC has been established. In conclusion, molecular analysis of EBC samples from patients with LC will allow us to explore the mechanisms underlying the successful diagnosis, therapy, and prevention of cancer and identify novel therapeutic targets.

## Data Availability

The original contributions presented in the study are included in the article/Supplementary Materials, further inquiries can be directed to the corresponding authors.

## References

[B1] Campbell P. J. Stephens P. J. Pleasance E. D. O'Meara S. Li H. Santarius T. (2008). Identification of somatically acquired rearrangements in cancer using genome-wide massively parallel paired-end sequencing. Nat. Genet. 40 (6), 722–729. 10.1038/ng.128 18438408 PMC2705838

[B2] Chen L. Han X. Hu Z. Chen L. (2019). The PVT1/miR-216b/Beclin-1 regulates cisplatin sensitivity of NSCLC cells via modulating autophagy and apoptosis. Cancer Chemother. Pharmacol. 83 (5), 921–931. 10.1007/s00280-019-03808-3 30859368

[B3] Crowley E. Di Nicolantonio F. Loupakis F. Bardelli A. (2013). Liquid biopsy: Monitoring cancer-genetics in the blood. Nat. Rev. Clin. Oncol. 10 (8), 472–484. 10.1038/nrclinonc.2013.110 23836314

[B4] Davis M. D. Montpetit A. Hunt J. (2012). Exhaled breath condensate: An overview. Immunol. Allergy Clin. North Am. 32 (3), 363–375. 10.1016/j.iac.2012.06.014 22877615 PMC3417047

[B5] Ferlay J. Colombet M. Soerjomataram I. Parkin D. M. Piñeros M. Znaor A. (2021). Cancer statistics for the year 2020: An overview. Int. J. Cancer 149 (4), 778–789. 10.1002/ijc.33588 33818764

[B6] Ferreira H. J. Esteller M. (2018). Non-coding RNAs, epigenetics, and cancer: Tying it all together. Cancer Metastasis Rev. 37 (1), 55–73. 10.1007/s10555-017-9715-8 29374363

[B7] Gutschner T. Diederichs S. (2012). The hallmarks of cancer: A long non-coding RNA point of view. RNA Biol. 9 (6), 703–719. 10.4161/rna.20481 22664915 PMC3495743

[B8] Hirsch F. R. Scagliotti G. V. Mulshine J. L. Kwon R. Curran W. J. Jr. Wu Y. L. (2017). Lung cancer: Current therapies and new targeted treatments. Lancet 389 (10066), 299–311. 10.1016/S0140-6736(16)30958-8 27574741

[B9] Hu X. Bao J. Wang Z. Zhang Z. Gu P. Tao F. (2016). The plasma lncRNA acting as fingerprint in non-small-cell lung cancer. Tumour Biol. 37 (3), 3497–3504. 10.1007/s13277-015-4023-9 26453113

[B10] Huarte M. (2015). The emerging role of lncRNAs in cancer. Nat. Med. 21 (11), 1253–1261. 10.1038/nm.3981 26540387

[B11] Jiang C. Li Y. Zhao Z. Lu J. Chen H. Ding N. (2016). Identifying and functionally characterizing tissue-specific and ubiquitously expressed human lncRNAs. Oncotarget 7 (6), 7120–7133. 10.18632/oncotarget.6859 26760768 PMC4872773

[B12] Li M. Zhang H. Zhao X. Yan L. Wang C. Li C. (2014). SPRY4-mediated ERK1/2 signaling inhibition abolishes 17beta-estradiol-induced cell growth in endometrial adenocarcinoma cell. Gynecol. Endocrinol. 30 (8), 600–604. 10.3109/09513590.2014.912264 24811094

[B13] Lung Cancer Survival Rates Lung cancer survival rates. American Cancer Society. Available from: https://www.cancer.org/cancer/lung-cancer/detection-diagnosis-staging/survival-rates.html. (Accessed November 11, 2022)

[B14] Nie F. Q. Sun M. Yang J. S. Xie M. Xu T. P. Xia R. (2015). Long noncoding RNA ANRIL promotes non-small cell lung cancer cell proliferation and inhibits apoptosis by silencing KLF2 and P21 expression. Mol. Cancer Ther. 14 (1), 268–277. 10.1158/1535-7163.MCT-14-0492 25504755

[B15] Pan L. J. Zhong T. F. Tang R. X. Li P. Dang Y. W. Huang S. N. (2015). Upregulation and clinicopathological significance of long non-coding NEAT1 RNA in NSCLC tissues. Asian Pac J. Cancer Prev. 16 (7), 2851–2855. 10.7314/apjcp.2015.16.7.2851 25854373

[B16] Rong F. Liu L. Zou C. Zeng J. Xu Y. (2020). MALAT1 promotes cell tumorigenicity through regulating miR-515-5p/EEF2 Axis in non-small cell lung cancer. Cancer Manag. Res. 12, 7691–7701. 10.2147/CMAR.S242425 32943920 PMC7468487

[B17] Schmitt A. M. Chang H. Y. (2016). Long noncoding RNAs in cancer pathways. Cancer Cell. 29 (4), 452–463. 10.1016/j.ccell.2016.03.010 27070700 PMC4831138

[B18] Shah D. R. Masters G. A. (2020). Precision medicine in lung cancer treatment. Surg. Oncol. Clin. N. Am. 29 (1), 15–21. 10.1016/j.soc.2019.08.002 31757310

[B19] Shroff G. S. de Groot P. M. Papadimitrakopoulou V. A. Truong M. T. Carter B. W. (2018). Targeted therapy and immunotherapy in the treatment of non-small cell lung cancer. Radiol. Clin. North Am. 56 (3), 485–495. 10.1016/j.rcl.2018.01.012 29622080

[B20] Smolarz B. Zadrozna-Nowak A. Romanowicz H. (2021). The role of lncRNA in the development of tumors, including breast cancer. Int. J. Mol. Sci. 22 (16). 10.3390/ijms22168427 PMC8395147 34445129

[B21] Sorber L. Zwaenepoel K. Deschoolmeester V. Van Schil P. E. Van Meerbeeck J. Lardon F. (2017). Circulating cell-free nucleic acids and platelets as a liquid biopsy in the provision of personalized therapy for lung cancer patients. Lung Cancer 107, 100–107. 10.1016/j.lungcan.2016.04.026 27180141

[B22] Sun C. Li S. Zhang F. Xi Y. Wang L. Bi Y. (2016). Long non-coding RNA NEAT1 promotes non-small cell lung cancer progression through regulation of miR-377-3p-E2F3 pathway. Oncotarget 7 (32), 51784–51814. 10.18632/oncotarget.10108 27351135 PMC5239515

[B23] Tetik Vardarli A. Pelit L. Aldag C. Korba K. Celebi C. Dizdas T. N. (2020). Concordance in molecular genetic analysis of tumour tissue, plasma, and exhaled breath condensate samples from lung cancer patients. J. Breath. Res. 14 (3), 036001. 10.1088/1752-7163/ab739b 32031993

[B24] Wang X. Cheng Z. Dai L. Jiang T. Li P. Jia L. (2021). LncRNA PVT1 facilitates proliferation, migration and invasion of NSCLC cells via miR-551b/FGFR1 Axis. Onco Targets Ther. 14, 3555–3565. 10.2147/OTT.S273794 34113122 PMC8180410

[B25] Weber D. G. Johnen G. Casjens S. Bryk O. Pesch B. Jockel K. H. (2013). Evaluation of long noncoding RNA MALAT1 as a candidate blood-based biomarker for the diagnosis of non-small cell lung cancer. BMC Res. Notes 6, 518. 10.1186/1756-0500-6-518 24313945 PMC4029199

[B26] Wei C. M. Zhao X. F. Qiu H. B. Ming Z. Liu K. Yan J. (2020). The long non-coding RNA PVT1/miR-145-5p/ITGB8 axis regulates cell proliferation, apoptosis, migration and invasion in non-small cell lung cancer cells. Neoplasma 67 (4), 802–812. 10.4149/neo_2020_190723N657 32202906

[B27] Yao X. Wang T. Sun M. Y. Yuming Y. Guixin D. Liu J. (2022). Diagnostic value of lncRNA HOTAIR as a biomarker for detecting and staging of non-small cell lung cancer. Biomarkers 27 (6), 526–533. 10.1080/1354750X.2022.2085799 35959801

[B28] You J. S. Jones P. A. (2012). Cancer genetics and epigenetics: Two sides of the same coin? Cancer Cell. 22 (1), 9–20. 10.1016/j.ccr.2012.06.008 22789535 PMC3396881

[B29] Zeng S. H. G. Xie J. H. Zeng Q. Y. Dai S. H. H. Wang Y. Wan X. M. (2021). lncRNA PVT1 promotes metastasis of non-small cell lung cancer through EZH2-mediated activation of hippo/NOTCH1 signaling pathways. Cell. J. 23 (1), 21–31. 10.22074/cellj.2021.7010 33650817 PMC7944120

[B30] Zhang R. Xia Y. Wang Z. Zheng J. Chen Y. Li X. (2017). Serum long non coding RNA MALAT-1 protected by exosomes is up-regulated and promotes cell proliferation and migration in non-small cell lung cancer. Biochem. Biophys. Res. Commun. 490 (2), 406–414. 10.1016/j.bbrc.2017.06.055 28623135

